# Treatment and prognosis of failure of first-line immunotherapy or recurrent autoimmune encephalitis patients with ofatumumab—a fully human anti-CD20 mAb

**DOI:** 10.3389/fneur.2025.1671481

**Published:** 2025-10-13

**Authors:** Yajing Liu, Yanbo Zhang, Zhijiao Song, Shuanghao Feng, Tongxin Cao, Hui Bu

**Affiliations:** ^1^Department of Neurology, The Second Hospital of Hebei Medical University, Shijiazhuang, Hebei, China; ^2^Key Laboratory of Clinical Neurology (Hebei Medical University), Ministry of Education, Shijiazhuang, Hebei, China; ^3^Neurological Laboratory of Hebei Province, Shijiazhuang, Hebei, China; ^4^Department of Geriatrics, Xiongan Xuanwu Hospital, Xiongan New Area, China

**Keywords:** Ofatumumab, failure of first-line immunotherapy autoimmune encephalitis, recurrent autoimmune encephalitis, CD20 mAb, B cells

## Abstract

**Background:**

Autoimmune encephalitis (AE) is a kind of encephalitis mediated by the autoimmune response. There is no uniform standard for immunotherapy of this disease, especially for failure of first-line immunotherapy or recurrent AE. Here, we report the case data of patients with failure of first-line immunotherapy or recurrent AE who were treated with ofatumumab (OFA).

**Methods:**

We retrospectively analyzed 18 patients with failure of first-line immunotherapy or recurrent adverse events treated with OFA. We collected general information, clinical manifestations, auxiliary examinations, treatment and prognosis, and adverse reactions. A retrospective analysis was conducted on these data, and the results were discussed in conjunction with a review of the relevant literature.

**Results:**

Among the 18 patients treated with OFA, significant improvements were observed in psychiatric symptoms (*p* = 0.005) and seizure frequency (*p* = 0.002). The CD20^+^ B cell levels declined most rapidly within the first week after the initial OFA injection and reached their lowest point at 1 month post-treatment. However, as the interval between doses increased, CD20^+^ B cell levels rebounded. In some patients who received repeated injections, CD20^+^ B cell levels were further reduced and maintained at a lower level. Both the modified Rankin Scale (mRS) and Clinical Assessment Scale for Autoimmune Encephalitis (CASE) scores showed statistically significant improvement post-OFA treatment (*p* < 0.01).

**Conclusion:**

Clinical data indicate that OFA demonstrates therapeutic efficacy in failure of first-line immunotherapy or recurrent AE, as evidenced by improved symptom control, decreased relapse rates, and an acceptable safety profile. However, this study is retrospective, and the clinical sample size is small, which may be biased. In the follow-up study, we will further expand the scale of case collection to further prove the efficacy and prognosis of OFA in treating AE patients.

## 1 Introduction

Autoimmune encephalitis (AE) refers to a class of encephalitis mediated by autoimmune disorder. Since the discovery of anti-N-methyl-D-aspartate receptor (NMDAR) encephalitis in 2007, a series of autoantibodies against neuronal cell surface or synaptic proteins have been discovered in AE patients (1). The “Chinese Consensus on Diagnosis and Treatment of Autoimmune Encephalitis” in 2022 defined recurrent AE is the recurrence of symptoms or aggravation of symptoms (mRS score increased by 1 point or more) after the symptoms have improved or stabilized for more than 2 months (2).

Currently, the treatments for AE include immunotherapies, symptom control therapies, supportive therapies and rehabilitation therapies. Immunotherapies include first-line, second-line and maintenance immunotherapy (3). First-line immunotherapy includes glucocorticoids, intravenous immunoglobulin (IVIG) and plasma exchange (PLEX). After first-line treatment, a steroid-sparing regimen or a tapering strategy of oral prednisone overlapping with azathioprine or mycophenolate mofetil (MMF) should be implemented to achieve sustained immunosuppression (3). The second-line immunotherapy is initiated when the severity of the disease increases and a poor response is observed to first-line immunotherapy. The second-line immunotherapy includes cyclophosphamide and anti-CD20 antibody therapy, such as rituximab (RTX). Although immunotherapy has developed to improve or stabilize the symptoms in most patients, about 40% of patients displayed poor prognosis post immunotherapy (4, 5). Recently studies revealed that AE patients who receive early immunotherapy have a better prognosis, suggesting the prognosis of AE is related to the timing of initiation of immunotherapy (6).

B-cell-mediated auto-immune response is the major immunopathogenic mechanism of AE, and targeting B cells with CD20 monoclonal antibodies is considered as a therapeutic strategy for AE. Anti-CD20 monoclonal antibodies are immunomodulating agents whose function is to trigger complement-mediated cytotoxicity, leading to depletion of CD20^+^ B lymphocytes (7). RTX is one of the anti-CD20 monoclonal antibodies (mAb) that has been proved effective in treating AE, but there are some controversies about adverse reactions and timing of RTX treatment (8).

To restrain the adverse effects of anti-CD20 antibody therapy, fully humanized anti-CD20 monoclonal antibodies have been developed in recent years. OFA is one of the fully humanized anti-CD20 antibodies, and displays fewer side effects than RTX (9). Currently, OFA is approved for the treatment of relapsing multiple sclerosis (10). There are some case reports of its use in AE patients, but lack of large sample studies (11).

To reveal the effectiveness and safety of OFA treatment in AE, 18 AE patients were treated with OFA in our study. The results show that OFA treatment ameliorated the symptoms of the failure of first-line immunotherapy or recurrent AE patients, as well as decreased the number of CD20^+^ B cells in the blood, suggesting OFA is effective and safe for the failure of first-line immunotherapy or recurrent AE therapy.

## 2 Materials and methods

### 2.1 Study design

This study was conducted as a single-center, retrospective cohort study. Patients who received subcutaneous injection of OFA at our hospital between September 2022 and October 2024 were identified through a review of the electronic medical record system. Due to the retrospective nature of the research, data collection was limited by the completeness and accuracy of the medical records, which may introduce information bias.

### 2.2 Population

The study recruited 20 Chinese patients diagnosed with failure of first-line immunotherapy or recurrent AE and treated with OFA. Data from 18 patients were retrospectively collected and analyzed, while 2 patients were excluded due to incomplete data or a lack of follow-up. Inclusion criteria: (1) Patients should meet the diagnostic criteria proposed in the “Chinese Consensus on Diagnosis and Treatment of Autoimmune Encephalitis” in 2022 (2); (2) We defined 1 cycle of first-line immunotherapy as 1,000 mg/d of methylprednisolone pulse for 5 days, 0.4 g/ kg/d of IVIg for 5 days, alone or combined. Failure of first-line immunotherapy was defined as the mRS score remaining at 4 or higher and/or no sustained improvement of the most prominent symptom, assessed by 2 experienced neurologists after 2–4 weeks from the treatment initiation; otherwise considered responded to first-line immunotherapy. Relapse of encephalitis was defined as symptom worsening or a new onset after at least 2 months of disease improvement or stabilization (12); (3) Received at least one subcutaneous injection of OFA; (4) This study has been approved by the Ethics Committee of the Second Hospital of Hebei Medical University (approval number: 2023-R605); (5) All the patients and their legal guardians who participated in this study have known the research contents and signed the informed consent form. Exclusion criteria: (1) Complicated with serious diseases: failure of important organs or systemic immune diseases; (2) Poor compliance: the patient could not cooperate with the study for personal reasons, or refused to provide clinical data and follow-up information; (3) Lost follow-up: unable to complete follow-up or lost follow-up during the study period. This study finally included 18 patients who met the standard for data analysis. Patients received subcutaneous injections of OFA 20 mg/w until the CD20^+^ B cell proportion decreased to below 1%. Thereafter, CD20 levels were monitored monthly, and additional injections were administered if the levels exceeded 1%. Some patients discontinued treatment after the initial phase due to financial constraints and did not receive subsequent therapy. OFA was initiated as sequential monotherapy following the discontinuation of prior immunosuppressive treatments due to inadequate response. Throughout the OFA treatment period, no concomitant immunomodulatory therapies were administered, allowing for evaluation of its independent therapeutic effect.

### 2.3 Outcome measures

All the patients involved in this study received at least one subcutaneous injection of OFA. Recurrence during the follow-up period was defined as the emergence or worsening of encephalitis symptoms after improvement or stabilization for at least 2 months (13), as judged by the treating neurologist based on overall clinical impression. Detailed clinical assessments and laboratory investigations were conducted at baseline and consecutive visits for each patient. The demographic data (age, gender, medical history) and clinical data (duration from onset to medication, presenting clinical features, types of antibodies) were collected. In addition, at baseline, the number and ratio of CD20^+^ B cells in peripheral blood were determined after 0 week (first visit), 1 week (second visit), 1 month (third visit), 2 months (fourth visit) and 6 months (fifth visit). In addition, modified Rankin Scale (mRS) and clinical assessment scale for autoimmune encephalitis (CASE) were used to evaluate the severity of the disease. The recurrence was recorded during the follow-up period. The main efficacy endpoint of this study was the case score after treatment or rapid symptom improvement. Secondary outcome measures included mRS score, CASE score, and recurrence during follow-up.

### 2.4 Safety assessment

Drug-related adverse events of patients from the initial medication to the whole medication period were recorded. These adverse reactions include injection-related reactions, local or systemic symptoms such as pain, fever, and headache at the injection site and infections, including lung infection, urinary system infection, and other systemic infections (such as bloodstream infection, central nervous system infection, etc.).

### 2.5 Statistical analysis

Use SPSS 25.0 software to make statistics and analysis on relevant data. Among them, the counting data is expressed by relative composition ratio (%) or rate (%); The measurement data were tested for normality by drawing a histogram and normal probability diagram (Q-Q diagram). The measurement data of normal distribution (including age, percentage of peripheral CD20^+^ B cells, mRS and CASE scores before and after medication) were expressed by mean standard deviation (SD). For the measurement data of non-normal distribution (including the time from onset to application of OFA treatment, etc.), the median (M) and interquartile interval (P25, P75) are used to describe it. Repeated measurement analysis of variance was used to compare mRS and CASE scores before and after medication. When *p* ≤ 0.05, the difference is considered statistically significant.

## 3 Results

### 3.1 General information

According to the above inclusion and exclusion criteria, this study included 18 patients with failure of first-line immunotherapy or recurrent AE who were hospitalized in the Department of Neurology of the Second Hospital of Hebei Medical University from September 2022 to October 2024 and received OFA treatment, including 12 males and 6 females. The onset age of the patients ranged from 16 to 77 years, with an average age of 38.06 ± 18.99 years.

Classification of different antibodies in AE patients: anti-NMDAR antibody was positive in 11 cases (61.1%), anti-GAD65 antibody was positive in 4 cases (22.2%), anti-LGI1 antibody was positive in 2 cases (11.1%), and anti-GABABR antibody was positive in 1 case (5.6%). Three patients (16.7%) with anti-NMDAR encephalitis were combined with anti-glial cell antibody (MOG), and one patient (5.6%) with anti-NMDAR encephalitis was combined with Glial Fibrillary Acidic Protein (GFAP). According to the types of diseases, 7 cases (38.9%) were failure of first-line immunotherapy and 11 cases (61.1%) were recurrent. Previous treatment: glucocorticoid pulse therapy in 14 cases (77.8%), intravenous injection of human immunoglobulin in 18 cases (100%), plasma exchange in 1 case (5.6%), mycophenolate mofetil in 6 cases (33.3%), intrathecal injection of methotrexate in 3 cases (16.7%) and rituximab in 3 cases (16.7%) (Table 1).

**Table 1:** Case characteristics of patients with autoimmune encephalitis.

Variables	Quantity (%)/median (interquartile interval)
Age at onset, y	38.06 ± 18.99
Sex	
Male	12 (66.7%)
Female	6 (33.3%)
Antibody type	
Anti-NMDAR antibody	11 (61.1%)
Anti-GAD65 antibody	4 (22.2%)
Anti-LGI1 antibody	2 (11.1%)
Anti-GABABR antibody	1 (5.6%)
Type of disease	
First onset	7 (38.9%)
failure of first-line immunotherapy	7 (38.9%)
Previous treatment	
GC	14 (77.8%)
IVIG	18 (100%)
PLEX	1 (5.6%)
MMF	6 (33.3%)
Intrathecal injection of MTX	3 (16.7%)
RTX	3 (16.7%)
Time from onset to OFA, d	18.00 (6.00, 29.25)
Symptoms	
Psychiatric symptoms	10 (55.6%)
Cognitive impairment	3 (16.7%)
Seizures	11 (61.1%)
Movement disorders	5 (27.8%)
Speech disorders	2 (11.1%)
Decline of consciousness level	1 (5.6%)
Autonomic symptoms	3 (16.7%)
Sleep disorder	1 (5.6%)
Blurred vision	2 (11.1%)
MRI	
Normal	9 (50.0%)
Abnormal	9 (50.0%)
EEG	
Normal	7 (38.9%)
Abnormal	11 (61.1%)

NMDAR, N-Methyl-D-Asparate Receptor; GAD65, Glutamate Decarboxylase 65; LGI1, Leucine Rich Glioma Inactivated 1; GABABR, Gamma Aminobutyric Acid Type B Receptor; GC, glucocorticosteroid; IVIG, intravenous immunoglobulin; PLEX, plasma exchange; MMF, mycophenolate mofetil; MTX, methotrexate; RTX, rituximab; MRI, magnetic resonance imaging; EEG, electroencephalogram.

### 3.2 Accessory examination

**Figure 1:**
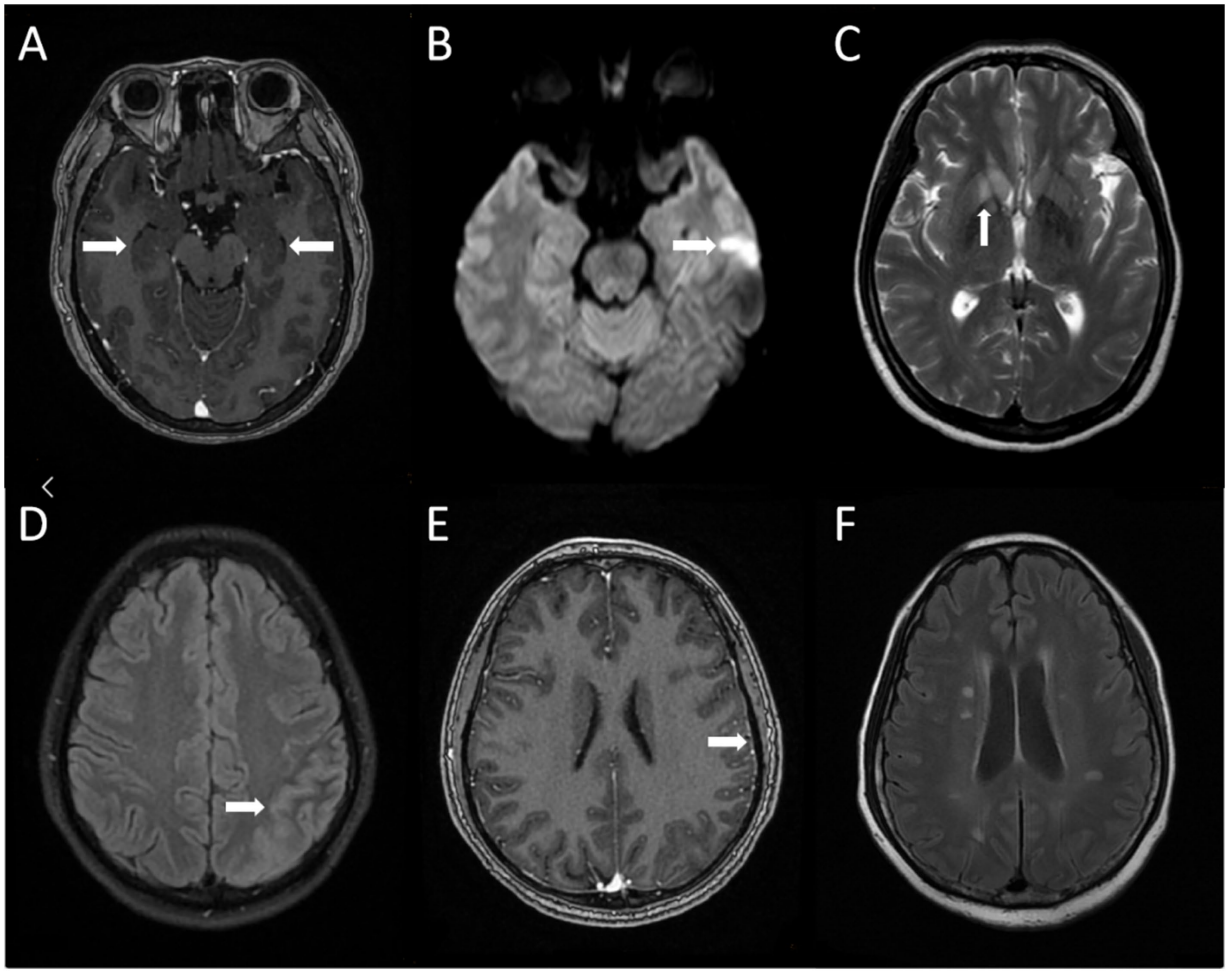
Brain MRI Characteristics of AE Patients. **(A)** Brain magnetic resonance enhanced scan in case 9 showed bilateral hippocampal swelling with abnormal signal; **(B,C)** brain magnetic resonance scan in case 10 showed abnormal signal of left temporal lobe and right basal ganglia; **(D,E)** brain magnetic resonance plain scan in case 13 showed the left temporal parietal lobe gyrus is swollen and the cerebral magnetic resonance enhanced scan in case 13 showed adjacent meninges are slightly thickened; **(F)** brain magnetic resonance plain scan in case 14 showed multiple abnormal signals in bilateral corona radiata.

Eighteen cases of AE were examined by MRI, and 9 cases (50.0%) had abnormal MRI findings, which could involve hippocampus (*n* = 4, 22.2%), insula (*n* = 3, 16.7%), basal ganglia (*n* = 2, 11.1%) and frontal lobe (*n* = 1, 5.6%) (Figure 1).

All 18 patients underwent EEG examination, including 7 cases with normal EEG (38.9%) and 11 cases with abnormal EEG (61.1%). Slow waves increased in 8 patients (44.4%) and epileptic waves in 7 patients (38.9%).

### 3.3 Efficacy of OFA treatment

**Figure 2:**
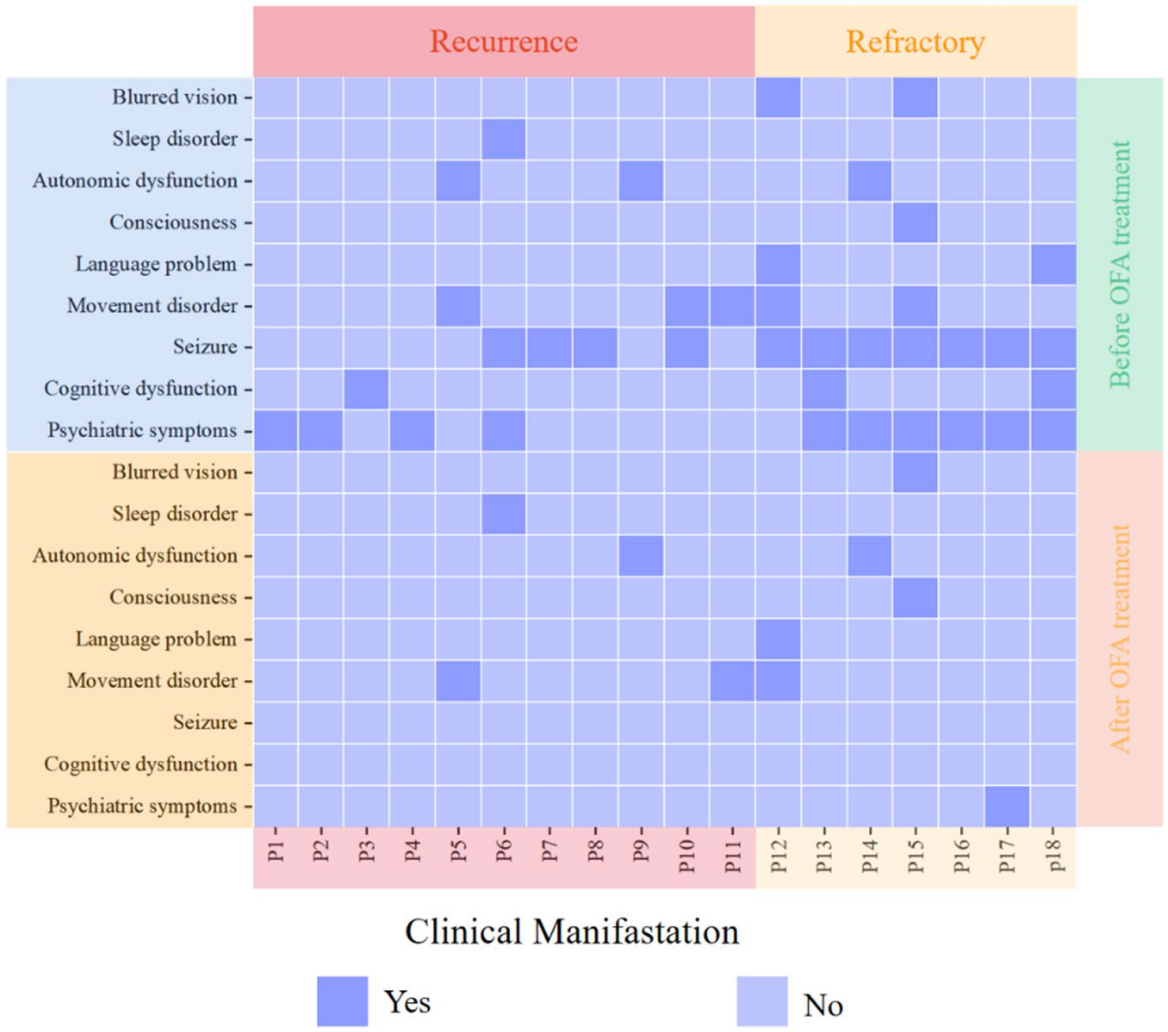
Alterations in clinical manifestations following OFA treatment.

During the follow-up, the symptoms of the patients were significantly improved after OFA treatment. All patients who had seizures had fewer seizures, 9 patients had improved mental symptoms, 3 patients had improved cognitive function, 2 patients had reduced motor disorder, 1 patient had reduced speech disorder, 1 patient had recovered autonomic nervous function, and 1 patient had improved blurred vision (Figure 2). Wilcoxon signed rank test showed that OFA treatment significantly improved the mental symptoms (*p* = 0.005) and seizures (*p* = 0.002) of AE patients. After OFA treatment, two patients with refractory mental symptoms and epilepsy have improved, but they still cannot fully return to normal life. The rest of the patients have not experienced disease recurrence.

**Figure 3:**
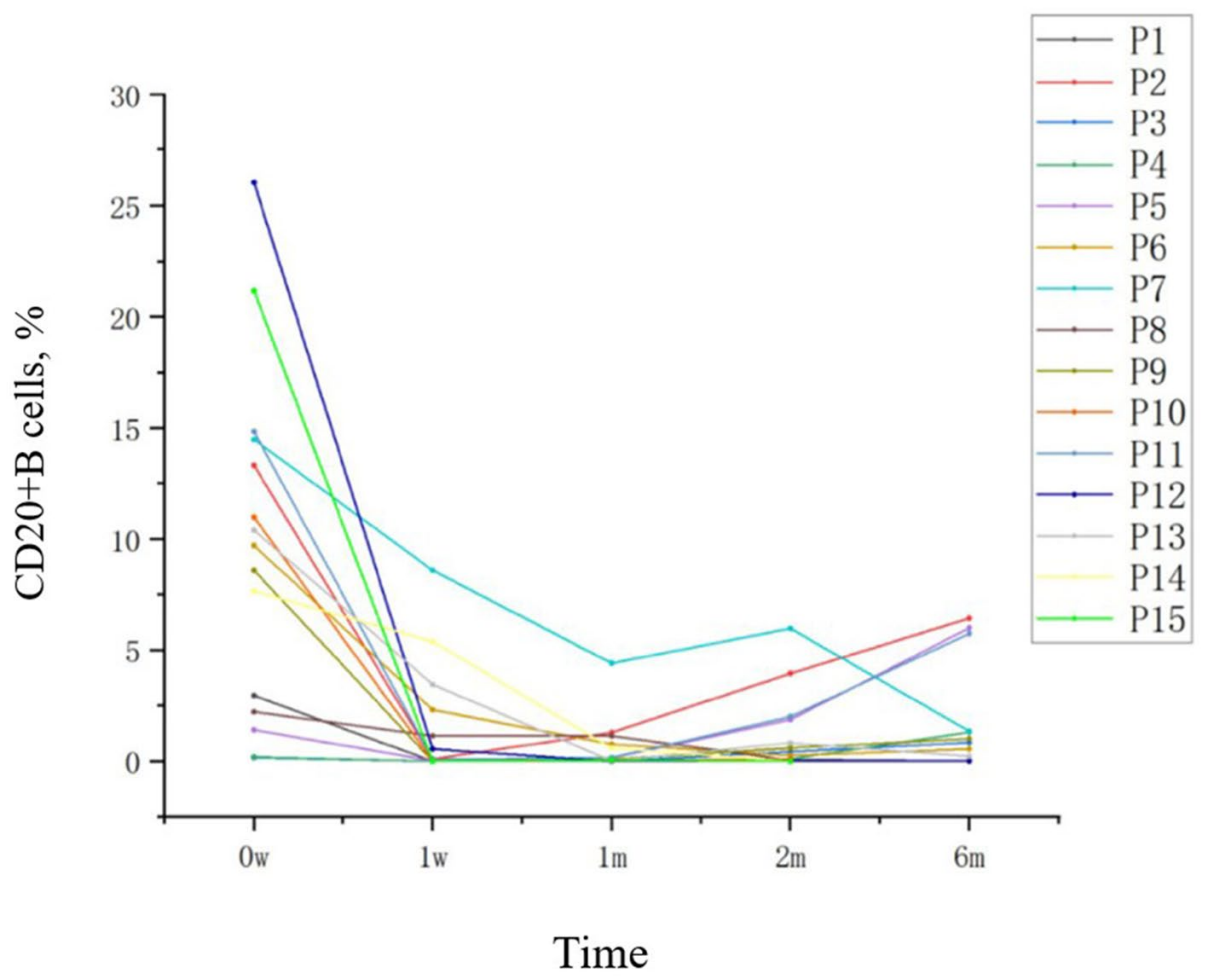
Trends in CD20^+^ B cell levels before and after OFA treatment.

The percentage of CD20^+^ B cells in the peripheral blood of patients with failure of first-line immunotherapy or recurrent AE treated with OFA decreased significantly (*p* < 0.05). The percentage of CD20^+^ B cells was the highest before treatment, with an average of 9.61 ± 7.65%. The recurrent AE patients were 6.40 ± 5.59%, and the failure of first-line immunotherapy AE patients were 16.03 ± 7.58%. After 1 week, it decreased significantly to 1.44 ± 2.54%. CD20^+^ B cell counts were significantly reduced from baseline by a mean of 8.17 points (95% CI: −12.46 to −3.88; *p* < 0.001). After 1 month, the percentage of CD20^+^ B cells decreased to the lowest, which was 0.58 ± 1.14%. After 2 months, the percentage of CD20^+^ B cells rebounded to 1.08 ± 1.74%. The percentage of CD20^+^ B cells gradually increased with the extension of medication time, and reached 1.81 ± 2.47% after 6 months of medication (Figure 3). During the follow-up, the percentage of CD20^+^ B cells in 7 patients continued to decrease and remained at a low level after repeated subcutaneous injection of OFA, which did not show the above trend.

**Figure 4:**
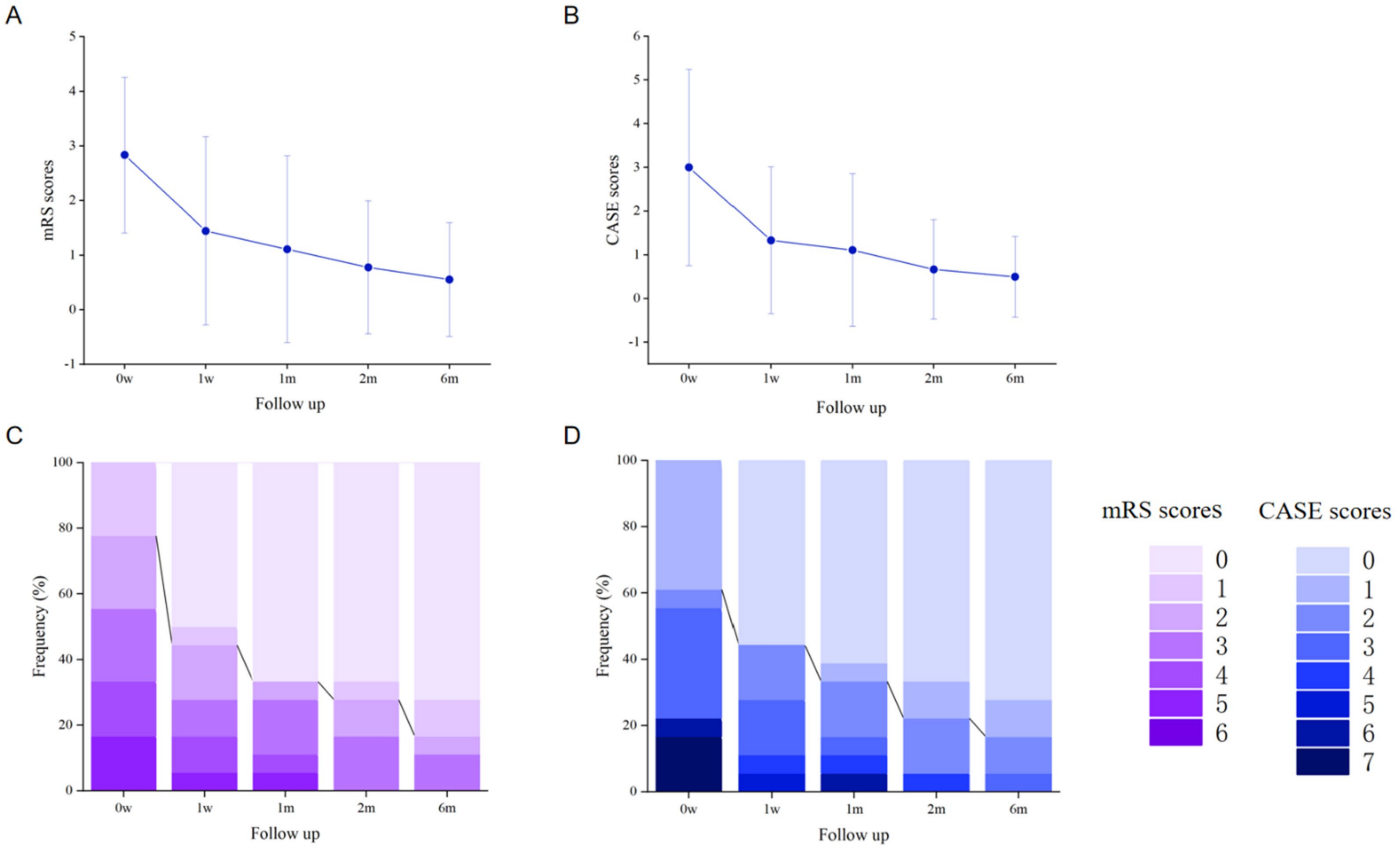
Changes of mRS and CASE scores in patients with failure of first-line immunotherapy or recurrent autoimmune encephalitis. **(A,B)** Comparison of the improvement trend of the mRS score and case score during the 6-month follow-up period. Error bars indicate the mean plus or minus standard deviation. **(C,D)** Functional outcomes during the first 6-month follow-up.

Eighteen patients were evaluated by mRS and CASE scores at the time of 0 w, 1 w, 1 m, 2 m and 6 m (Table 2). The mRS and CASE scores were normally distributed at all time points, and gradually decreased with the extension of medication time (Figures 4A,B). During the 6-month follow-up, the proportion of patients with an mRS score ≤2 continued to increase (Figures 4C,D). After OFA injection, there were significant differences in mRS scores between 0 w and 1 w (*p* < 0.01), 1 m (*p* < 0.01), 2 m (*p* < 0.01) and 6 m (*p* < 0.01). The CASE scores of 0 w are significantly different from those of 1w (*p* < 0.01), 1 m (*p* < 0.01), 2 m (*p* < 0.01) and 6 m (*p* < 0.01) (Table 3). Following 1 week of OFA treatment, a significant improvement in mRS scores from baseline was observed (mean change: −1.39; 95% CI: −1.93 to −0.86; *p* < 0.001). The most substantial improvement in mRS scores occurred after 6 months of OFA therapy (mean change: −2.28; 95% CI: −2.56 to −1.60; *p* < 0.001). Similarly, CASE scores showed significant improvement after 1 week of OFA treatment (mean change: −2.28; 95% CI: −2.96 to −1.60; *p* < 0.001). The greatest improvement in CASE scores was also evident at the 6-month time point (mean change: −2.50; 95% CI: −3.40 to −1.60; *p* < 0.001). At the 6-month follow-up, the mean mRS score was 0 in the group with CD20^+^ B cells ≥1% and 1.55 in the group with CD20^+^ B cells <1%. No significant difference was observed between the two groups (*p* ≥ 0.05).

**Table 2:** Comparison of mRS, CASE scores before and after OFA treatment ( $\bar{X}$ ±*s*).

Score	Before OFA	1w	1 m	2 m	6 m
mRS	2.83 ± 1.42	1.44 ± 1.72	1.11 ± 1.71	0.78 ± 1.21	0.56 ± 1.04
CASE	3.00 ± 2.24	1.33 ± 1.68	1.11 ± 1.74	0.67 ± 1.13	0.50 ± 0.92

**Table 3:** Analysis of variance table for mRS, CASE scores before and after OFA treatment using a pre-post study design.

Sources of variation	*SS*	*df*	*MS*	*F*	*P*
mRS					
Between-group variance	58.044	4	14.511	6.919	0.000*
Within-group variance	178.278	85	2.097		
Total variation	236.322	89			
CASE					
Between-group variance	71.378	4	17.844	6.824	0.000*
Within-group variance	222.278	85	2.615		
Total variation	293.656	89			

*P<0.01.

### 3.4 Adverse reactions and safety of OFA

Among the 18 patients who used OFA, one patient had symptoms of fever and pulmonary infection, and the infection was controlled after antibiotics and symptomatic treatment. Two patients had intermittent pain in finger joints and knee joints; One patient felt obvious muscle soreness and relieved himself after rest. Other patients did not have injection-related reactions, lymphopenia, thrombocytopenia and other adverse reactions. No serious adverse reactions such as disability and death occurred in all patients.

## 4 Discussion

AE is a new class of immune-mediated diseases in the central nervous system (CNS), characterized by pathogenic autoantibodies directed against neuronal surface or intracellular proteins (14). Traditional first-line therapy for AE includes corticosteroids, IVIg, and PLEX, which are limited by poor pathogenesis specificity and short duration of maintenance therapy. Moreover, oral prednisone for bridging and steroid-sparing and azathioprine or mycophenolate mofetil for sustained immunosuppression have the drawbacks of poor specificity, complexity and variety of treatment regimens as well as persistent adverse effects (3). In addition, about 40% of patients have a poor prognosis or remain refractory after immunotherapy up to date (4, 5). It’s recommended to early initiation of immunotherapy early and the application of second-line agents based on systematic evaluations of AE, which has displayed better functional outcomes and lower relapse rates with manageable side effects recently (15). In the “Expert Consensus on Diagnosis and Treatment of Autoimmune Encephalopathy in China” published in 2022, it is emphasized that second-line immunotherapy includes anti-CD20 monoclonal antibodies and intravenous cyclophosphamide, which is mainly used for refractory or Recurrent patients with poor first-line immunotherapy (2).

During the course of AE disease, the expression of CD20 is gradually increased in B cells, and it is consistently expressed at high levels on the surface of antibody-specific memory B cells and plasmablasts (16). Moreover, activated CD20^+^ B cells could present the same specific antigens to T lymphocytes in association with MHC molecules in the presence of multiple co-stimulatory factors, thereby promoting T cell activation and differentiation. Subsequently, these T cells produce a variety of cytokines and chemokines to regulate the maturation and migration of peripheral immune effector cells (such as helper T lymphocytes, CD8^+^ T cells, and myeloid cells). These cells can also secrete a range of pro-inflammatory mediators that induce neuroinflammation within the CNS parenchyma (17). Therefore, B-cell depleting drugs can have beneficial effects on AE treatment by inhibiting neuroinflammation through targeting CD20^+^ B lymphocytes. RTX is the first licensed anti-CD20 mAb, which has displayed positive effects in treatment for neuroimmunological diseases (18). It mainly targets the outer ring epitopes of large cells (19, 20), mainly through complement-dependent cytotoxicity (CDC), antibody-dependent cell-mediated cytotoxicity (ADCC) and antibody-dependent cell-mediated phagocytosis (ADCP) leading to CD20^+^ B cell depletion. Phase II/III OLYMPUS experiment reported that CD19^+^ B cells were rapidly depleted and the depletion rate exceeded 95% 2 weeks after the first 1,000 mg infusion (21). Due to RTX being a human-mouse chimeric mAb, the overall adverse reaction rate of RTX intravenous infusion is 4.2%, suggesting that a more effective and safer anti-CD20 mAb is needed (22).

OFA is a fully human mAb that binds to two distinct regions within the large and small extracellular loops of CD20. OFA achieves deep depletion of B cells mainly through CDC and ADCC (23, 24). Compared to RTX, OFA is less immunogenic and has a favorable safety profile. The rate of serious infections in MS patients treated with OFA is 2.5%, while the rate of serious infections in MS patients treated with RTX is 4.5% (25, 26). In addition, the administration time of OFA is less than that of RTX during maintenance therapy, which results in a lower rate of end-of-cycle relapses. Subcutaneous delivery methods allow relatively stable patients to rapidly self-administer their medications at home, which can reduce the length of hospitalization and thus the financial burden caused by hospitalization (27). And compared with bortezomib, tocilizumab, CAR-T and other emerging therapies in failure of first-line immunotherapy AE, OFA is safer in the application of failure of first-line immunotherapy autoimmune encephalitis.

The issue of blood–brain barrier (BBB) penetration by OFA in AE is of significant importance. Similar to RTX, OFA is a large monoclonal antibody with limited passive diffusion capacity across an intact BBB. However, in AE, the disruption of the BBB significantly enhances the penetration of large antibody-based therapeutics. OFA is administered subcutaneously, enabling sustained and low-level drug concentrations in the serum. This “low and steady” pharmacokinetic profile may facilitate gradual and continuous penetration of the antibody through impaired regions of the inflamed BBB via mechanisms such as paracellular leakage or transcytosis. This stands in contrast to the pronounced peak-and-trough concentration fluctuations associated with intravenous RTX administration. Notably, analyses from the Hauser study demonstrated complete depletion of B cells in the cerebrospinal fluid of multiple sclerosis patients treated with OFA. This provides compelling evidence that OFA not penetrates the central nervous system, but also exerts meaningful biological effects within it (25).

In this study, OFA can significantly improve mental symptoms and seizures (*p* < 0.05), and the proportion of patients with mRS score ≤2 continues to increase, which proves that OFA can effectively improve the clinical symptoms of failure of first-line immunotherapy or recurrent AE patients with poor first-line immunotherapy. After the first injection of OFA, the level of CD20^+^ B cells in patients’ peripheral blood decreased rapidly. The decline was the fastest in 1 week, and reached the lowest value in 1 month, and then the cell level rose. Some patients can continue to decrease and maintain at a low level after repeated injections. At regular follow-up, there was a continuous downward trend in the CASE score and mRS score. A comparison of the mean mRS scores at 6 months between the group with CD20^+^ B cells ≥1% and the group with CD20^+^ B cells <1% revealed no statistically significant difference (*p* ≥ 0.05). This suggests that continued OFA treatment did not exert a significant influence on the 6-month prognostic outcomes. Previous case series reported that patients with failure of first-line immunotherapy or recurrent AE stopped taking OFA for two or three consecutive times within 3 weeks after the symptoms improved. During the three-month follow-up, mRS and Case scores could be improved continuously, and there was no symptom deterioration or recurrence (28). During the follow-up of 6 months, the remission rate of OFA in the treatment of failure of first-line immunotherapy or recurrent AE was 88.89%. No relapse has occurred in regular follow-up after application of OFA.

The prognosis of two failure of first-line immunotherapy AE patients in this cohort is relatively poor, and both patients are double antibody positive. One patient with positive anti-NMDAR and MOG double antibodies continued to get worse after the first OFA injection, and the level of CD20^+^ B cells decreased slowly (only by 2.28%) after 1 week of medication. After three consecutive OFA applications within 3 weeks, B cells were deeply exhausted (down to 0.6%), but the improvement of clinical symptoms still lagged. This suggests that the synergistic pathogenic mechanism mediated by double antibodies may involve more complex T/B cell interaction, and it is necessary to pay attention to the influence of antibody types on therapeutic response.

The fever and lung infection of two patients in this study recovered with antibiotics and symptomatic treatment, and one patient had muscle soreness and was relieved after rest, which confirmed that OFA was safe in the treatment of AE patients. Two patients who had been on long-term mycophenolate mofetil (MMF) therapy experienced arthralgia. This symptom had been intermittently present even prior to initiation of OFA treatment. The timeline did not show a clear correlation with OFA administration, and MMF is a known contributor to musculoskeletal symptoms. Therefore, this event was judged to be unrelated to OFA.

Our study had several limitations. This study is retrospective and the sample size is moderate, which carries inherent risks of biases such as selection bias and information bias. These limitations may affect the generalizability of our findings. Although we minimized confounding by administering OFA as sequential therapy following prior treatment failure, it remains impossible to fully exclude the influence of unmeasured confounding factors, as would be achievable in a randomized controlled trial. Nevertheless, the observed “treatment failure-response” pattern, together with the significant clinical improvements seen during OFA monotherapy, strongly suggests a therapeutic benefit of the agent. Furthermore, the absence of a concurrent control group prevents us from drawing causal conclusions; only associations between variables can be reported. Although AE patients with different antibodies exhibit distinct clinical manifestations, this study did not stratify the analysis of treatment efficacy by antibody type, which may obscure the underlying heterogeneity. Due to the lack of internal RTX control group, the comparison with literature data has limitations.

## 5 Conclusion

Our report formulated a novel exploration of AE treatment. OFA is relatively effective and safe for the treatment of AE. In cases where patients with AE are failure of first-line immunotherapy, OFA may represent a potential therapeutic option. However, larger-scale, prospective, long-term real-world studies are warranted to further substantiate its efficacy and long-term outcomes in the AE population.
